# Artificial Intelligence in Intraoperative Imaging and Navigation for Spine Surgery: A Narrative Review

**DOI:** 10.3390/jcm15072779

**Published:** 2026-04-07

**Authors:** Mina Girgis, Allison Kelliher, Michael S. Pheasant, Alex Tang, Siddharth Badve, Tan Chen

**Affiliations:** 1 Geisinger Northeast Orthopaedic Surgery Residency, Wilkes-Barre, PA 18702, USA; 2 Department of Orthopaedic Surgery, Geisinger Medical Center, Danville, PA 17822, USA; 3 Department of Orthopaedic Spine Surgery, Inova Orthopedics—Springfield, Alexandria, VA 22310, USA

**Keywords:** artificial intelligence, navigation, intraoperative imaging

## Abstract

Artificial intelligence (AI) is increasingly transforming spine surgery, with expanding applications in diagnostics, intraoperative imaging, and surgical navigation. As the field advances toward greater precision and safety, machine learning (ML) and deep learning technologies are being integrated to augment surgeon expertise and optimize operative workflows. In particular, AI-driven innovations in image acquisition and navigation are reshaping intraoperative decision-making and technical execution. This narrative review provides an overview of AI applications relevant to intraoperative imaging and navigation in spine surgery. We begin by defining key concepts in AI, ML, and deep learning and briefly outline the historical evolution of AI within spine practice. We then examine current capabilities in image recognition and automated pathology detection, emphasizing their clinical relevance. Given the central role of imaging accuracy in modern navigation-assisted procedures, we review conventional acquisition platforms, including intraoperative computed tomography (CT) systems (e.g., O-arm, GE, Airo), surface-based registration to preoperative CT (Stryker, Medtronic), and optical surface mapping technologies (e.g., 7D Surgical). Emerging AI-optimized advancements are subsequently discussed, including low-dose intraoperative CT protocols, expanded scan windows, metal artifact reduction algorithms, integration of 2D fluoroscopy with preoperative CT datasets, and 3D reconstruction derived from 2D imaging. These developments aim to improve image quality, reduce radiation exposure, and enhance navigational accuracy. By synthesizing current evidence and technological progress, this review highlights how AI-enhanced imaging systems are redefining intraoperative spine surgery and shaping the future of precision-based care. The primary purpose of this review is to outline the applications of AI and its potential for perioperative and intraoperative optimization, including radiation exposure reduction, workflow streamlining, preoperative planning, robot-assisted surgery, and navigation. The secondary purpose is to define AI, machine learning, and deep learning within the medical context, describe image and pathology recognition, and provide a historical overview of AI in orthopedic spine surgery.

## 1. Background/Objectives

### 1.1. AI and AI-Adjacent Technology Background

Artificial intelligence (AI) is an advancing field with the potential to revolutionize many aspects of spine surgery. Spine surgery has continuously evolved across multiple domains of clinical practice, including diagnostics, surgical approaches, procedures, and instrumentation, ultimately striving to provide higher-quality, safer, and more accurate patient care. Over the past decade, AI has become increasingly integrated into the surgical field, enabling surgeons to augment their expertise through rapidly advancing technological innovations [1].

Conventional intraoperative imaging in spine surgery involves significant trade-offs. Intraoperative CT systems, while highly accurate, are bulky, expensive, and expose patients and operating room personnel to ionizing radiation [2,3]. Fluoroscopy-based techniques are more accessible but provide only planar two-dimensional feedback, limiting spatial understanding of complex pedicle anatomy [4]. Surface registration to preoperative CT reduces intraoperative radiation but may sacrifice accuracy when anatomy is distorted by deformity or prior surgery [5,6]. These limitations—radiation burden, workflow disruption, cost, and spatial resolution—represent the central constraints around which modern imaging innovation has been organized.

This review addresses the following argument: that AI is progressively shifting intraoperative spine imaging from a passive acquisition paradigm—where surgeons interpret static images—toward an active decision-support framework in which computational systems reconstruct, enhance, and contextualize imaging data in real time. We examine how AI-enabled technologies, including low-dose reconstruction algorithms, 3D synthesis from 2D fluoroscopy, synthetic CT generation from MRI, and computer-vision-based registration, directly address the limitations of conventional platforms. We also identify where the evidence remains preliminary, where clinical validation is lacking, and what questions remain unresolved as these systems approach widespread adoption. The primary purpose of this review is to outline the applications of AI and its potential for perioperative and intraoperative optimization, including radiation exposure reduction, workflow streamlining, preoperative planning, robot-assisted surgery, and navigation. The secondary purpose is to define AI, machine learning, and deep learning within the medical context, describe image and pathology recognition, and provide a historical overview of AI in orthopedic spine surgery.

### 1.2. Explanation of the Fields of AI, Machine Learning, and Deep Learning

Artificial intelligence (AI) is an umbrella term describing computer systems designed to perform tasks that typically require human intelligence. Broadly, AI encompasses methods that replicate or augment human cognitive processes to enhance patient assessment, workflow efficiency, and data interpretation [7].

Machine learning (ML), a major branch of AI, focuses on building systems that learn from data rather than relying solely on predefined rules. As models are exposed to larger datasets, they identify patterns and iteratively refine their performance. ML is commonly categorized as supervised or unsupervised learning. Supervised learning uses labeled datasets to train models to predict specific outcomes, whereas unsupervised learning analyzes unlabeled data to identify underlying structure, such as clusters or variable associations [7].

In orthopedic spine surgery, ML algorithms analyze large datasets—including imaging, demographic information, comorbidities, operative variables, and patient-reported outcomes—to generate predictive or classification models. Common techniques include logistic regression, decision trees and random forests, support vector machines, Bayesian networks, and neural networks, each offering trade-offs between predictive performance and interpretability [8].

These models have applications across the preoperative, intraoperative, and postoperative settings. Preoperatively, ML supports diagnostic accuracy and clinical decision-making through analysis of large imaging datasets. Examples include automated measurement of radiographic angles and injury detection on plain radiographs, fracture classification on CT, opportunistic osteoporosis estimation, and automated grading of degenerative pathology, stenosis, and spondylolisthesis on MRI [9,10,11,12,13]. Intraoperatively, ML enhances image-guided assistance and navigation. Systems such as the 7D Surgical platform reconstruct preoperative CT scans into intraoperative three-dimensional spinal topography, improving workflow efficiency and achieving screw placement accuracy comparable to freehand techniques while reducing radiation exposure [14]. Similarly, the X23D system converts two-dimensional fluoroscopic images into three-dimensional reconstructions, enabling segmentation and pedicle trajectory identification that dynamically account for intraoperative changes [15]. Postoperatively, ML models are used to predict clinical outcomes, complications, readmission, and reoperation rates across diverse spine pathologies, including tumor, infection, deformity, and degenerative disease [8].

**Figure 1:**
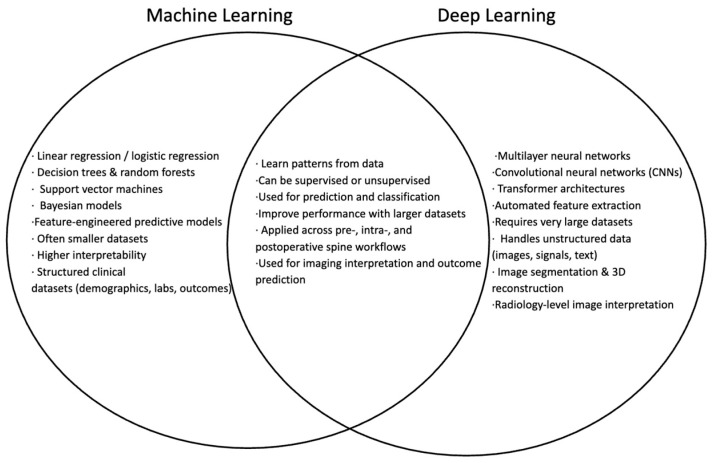
Demonstration of crossover between Machine Learning and Deep Learning. Conceptual comparison of machine learning and deep learning within artificial intelligence as applied to spine surgery. Traditional machine learning methods typically utilize structured clinical datasets and engineered features for prediction and classification tasks, whereas deep learning employs multilayer neural networks capable of automatically extracting features from large unstructured datasets such as medical imaging. The overlap highlights shared capabilities including pattern recognition, supervised or unsupervised learning, and applications in imaging interpretation and clinical outcome prediction across the perioperative spine surgery workflow.

Deep learning (DL) is a specialized subset of ML that employs multilayered artificial neural networks to model complex, nonlinear relationships within large datasets. These networks may contain dozens to thousands of layers, loosely mimicking the interconnected processing architecture of the human brain. While DL can be applied in supervised or unsupervised settings, its primary strength lies in analyzing high-dimensional and unstructured data. DL models are particularly effective in image segmentation, signal analysis (such as electromyography or neuromonitoring data), and natural language processing for extracting clinically meaningful information from operative notes and medical records. Owing to their scalability and accuracy, DL approaches have become central to modern medical AI and are increasingly integrated into imaging, navigation, and decision-support systems in spine surgery [7] (Figure 1).

## 2. Methods

This study was conducted as a narrative review of artificial intelligence applications in intraoperative imaging and navigation for spine surgery. Topics and technologies included in the review were predetermined by the senior author based on clinical relevance and commonly used or emerging platforms in spine surgery.

For each topic and technology identified, literature searches were performed to identify representative studies and supporting evidence. Searches were conducted using PubMed and Ovid MEDLINE databases. When peer-reviewed literature describing specific technologies was limited, additional information was obtained through targeted internet searches to identify manufacturer documentation, publicly available technical descriptions, and regulatory information such as U.S. Food and Drug Administration (FDA) clearance documentation.

Because the purpose of this review was to provide a focused overview of selected technologies and concepts rather than to systematically evaluate all available literature, a formal systematic review methodology and PRISMA-guided screening process were not performed.

## 3. Results

### 3.1. AI and AI-Adjacent Technology in Image Interpretation and Diagnostics

There has been significant progress in applying artificial intelligence (AI) to image recognition and diagnostic tasks in spine surgery, with the goal of supporting, rather than replacing, orthopaedic spine surgeons in evaluating and managing spinal conditions. In 2000, Sanders et al. used a supervised artificial neural network to automatically score patient pain drawings and classify them into five categories: benign back pain, herniated nucleus pulposus, spinal stenosis, serious underlying disorders, and psychogenic regional pain disturbance [16]. Their model was a shallow backpropagation neural network trained on labeled data, typical of early machine-learning approaches. Although the model achieved only moderate sensitivity (49%), its performance was comparable to physician experts and early statistical methods, demonstrating that even early supervised AI systems could extract meaningful diagnostic patterns from patient-reported data with the broader goal of supporting triage decision-making [16].

Building on these early efforts, Veronezi et al. in 2011 developed an AI-based system to identify lumbar spine osteoarthritis using lateral radiographs [17]. The authors implemented a hybrid neural network combining a Kohonen self-organizing map (an unsupervised feature-extraction method) with a multilayer perceptron classifier (a supervised neural network). After pre-processing 206 digital radiographs and training on 68 images, the model achieved moderate performance, with an accuracy of 62.85%, sensitivity of 65.71%, and specificity of 60%. This work demonstrated that early neural networks could identify clinically meaningful radiographic features, such as osteophytes, marking an important step toward automated imaging analysis in spine surgery [17].

More recently, deep-learning techniques have substantially advanced imaging-based diagnostics. In 2021, Pan et al. applied a multistage deep-learning framework to lumbar MRI, combining automated vertebral and disc localization with a convolutional neural network (CNN) for classification [18]. The system diagnosed disc bulge and herniation on axial MRI with accuracies ranging from 84.2% to 92.7% across intervertebral levels. All images were labelled by radiologists, and the supervised CNN reliably differentiated normal discs from bulges and herniations [18]. Similarly, Hallinan et al. developed a fully supervised deep-learning model to detect and grade lumbar spinal stenosis at the central canal, lateral recesses, and neural foramina [19]. Their system automatically identified regions of interest and used a custom CNN to classify stenosis as normal, mild, moderate, or severe. Across internal and external validation cohorts, the model demonstrated high sensitivity and specificity, with agreement comparable to subspecialty radiologists, supporting its role as a semiautomated assistive tool in clinical reporting [19].

Beyond degenerative pathology, AI has also demonstrated utility in trauma imaging. Machine-learning-based radiographic recognition models offer efficient, accurate, and reliable diagnostic support [10]. In 2022, Doerr et al. developed and validated a deep-learning model using an R-CNN architecture to automatically analyze thoracolumbar CT scans and predict two key components of the TLICS score: injury morphology and posterior ligamentous complex (PLC) integrity [11]. Morphology was predicted with 95.1% accuracy and PLC integrity with 86.8% accuracy. These findings suggest more efficient and consistent TLICS scoring in emergency settings, potentially reducing reliance on MRI and streamlining surgical triage for patients with thoracolumbar injury [11].

As AI continues to evolve within spine surgery, its predictive capacity and diagnostic performance are steadily improving. Nonetheless, current models remain limited by concerns related to data privacy, dataset quality, cybersecurity, liability, and generalizability across institutions and surgical practices. Despite these challenges, AI holds substantial promise in reducing diagnostic burden, enhancing efficiency, and streamlining workflow in spine surgery, with increasingly robust evidence supporting its clinical utility.

### 3.2. Importance of Image Acquisition and Navigation in Spine Surgery: Conventional Methods of Acquisition

While early AI work in spine focused on diagnostic classification and radiologic interpretation, intraoperative imaging and navigation represent a distinct and rapidly evolving domain where accuracy, workflow, and radiation exposure are central constraints.

### 3.3. AI-Enhanced and AI-Adjacent Intraoperative Imaging Platforms

#### 3.3.1. Intraoperative CT (O-Arm, GE, Airo)

Intraoperative CT systems, including the Medtronic O-arm, GE OEC 3D, and Airo True CT Mobile platforms, provide real-time three-dimensional imaging that enables highly precise pedicle screw and cage placement and assists in achieving appropriate decompression. In a study by Miller et al., screw trajectories planned using the O-arm navigation system were nearly identical to actual screw positions confirmed on intraoperative CT, with an average deviation of approximately 2° in both the axial and sagittal planes and excellent reliability [20]. Accuracy was maintained even when screws were placed several vertebral levels away from the reference frame, and only 3 of 240 screws (1.25%) required intraoperative revision. Notably, no neurologic, visceral, or vascular complications were attributed to screw placement. Collectively, these findings support the reliability of O-arm-based navigation, allowing surgeons to identify and correct malposition in real time and reduce clinically significant screw misplacement compared with traditional freehand techniques [20].

Similarly, in a prospective series of complex spinal deformity cases, Rajasekaran et al. evaluated pedicle screw placement using the AIRO mobile intraoperative CT navigation system and reported a 96.2% accuracy rate after accounting for planned in–out–in trajectories [21]. This high level of accuracy was achieved despite severely distorted pedicle anatomy and curves exceeding 60°. The authors also demonstrated efficient workflow, with a mean screw insertion time of 1.76 min, further supporting the reliability of intraoperative CT-based navigation in technically demanding deformity surgery.

The GE OEC 3D cone-beam CT system provides intraoperative two- and three-dimensional imaging with a large field of view and has been described as a workflow-efficient tool for routine verification of implant positioning. However, published data evaluating its pedicle screw placement accuracy remain limited, with most available evidence consisting of clinical experience reports rather than peer-reviewed validation studies. Although the OEC 3D system may facilitate intraoperative instrumentation assessment and potentially reduce the need for postoperative CT imaging, formal validation demonstrating accuracy comparable to established intraoperative CT platforms has not yet been reported [22]. In contrast, conventional two-dimensional fluoroscopy remains widely used due to its availability and lower cost; however, its reliance on planar imaging limits comprehensive assessment of complex three-dimensional pedicle anatomy, particularly in deformity surgery.

In a 10-year study by Costa et al. involving 2020 patients and 11,144 pedicle screws placed using an intraoperative CT-guided navigation system, an overall accuracy rate of 98.5% was reported [23]. The authors concluded that image-guided navigation provides excellent results, nearly eliminating the need for reoperation due to malpositioned instrumentation (screws, plates, or cages) or inadequate decompression. However, they emphasized that such technology cannot replace the surgical skill, experience, and anatomical knowledge required for successful outcomes [23]. Across multiple investigations, intraoperative CT combined with navigation-guided techniques has consistently demonstrated improvements in pedicle screw safety and accuracy. These systems allow real-time verification and correction of instrumentation, substantially reducing the likelihood of revision surgery for malposition.

Similarly, Zausinger et al. conducted a prospective interventional case series of 94 patients evaluating intraoperative CT for image acquisition following surgical positioning and navigation, with a repeat CT scan performed to assess final screw accuracy and adequacy of decompression [24]. Navigation accuracy was reported at 1 mm according to Odom criteria. The rate of major cortical breach (>2 mm) was 4.8%, while minor breaches occurred in 15.7% of screws. Immediate intraoperative correction was required in 2.4% of cases, and no reoperations were necessary due to implant malposition. The overall revision rate was 8.5%, attributable solely to cerebrospinal fluid leaks or wound-related complications. All patients demonstrated clinical improvement at three months.

With respect to workflow, preoperative scanning and data transfer required an average of 14 min, and intraoperative scanning resulted in a mean interruption of nine minutes. These findings suggest that CT-based navigation significantly enhances pedicle screw accuracy and safety while causing minimal disruption to operative workflow [24].

#### 3.3.2. Expanded Scan Window (O Arm)

Continued advances in O-arm technology have further enhanced operating room workflow, including the development of expanded scan window capabilities. The latest generation O-arm system (Medtronic) incorporates a 3D Long Scan (3DLS) mode that significantly increases the intraoperative imaging field of view. This mode allows volumetric three-dimensional imaging of up to approximately 43.8 cm and provides up to 2.7 times the navigable scan length of a standard 3D acquisition, enabling surgeons to visualize and navigate across a substantially larger spinal segment during a single registration [25].

This expanded imaging window is particularly advantageous for long-segment constructs and complex deformity corrections, where conventional limited-volume scans often require multiple acquisitions or patient repositioning. Zhang et al. evaluated long-length intraoperative imaging in the setting of extended spinal constructs using both phantom and cadaveric models [26]. The authors reported longitudinal coverage of 50–64 cm with a single long-length scan, providing a field of view of approximately 40 × 64 cm^2^, depending on patient positioning. Long-length scanning produced high-resolution imaging compared with alternative reconstruction techniques and enabled visualization of 12–16 vertebral levels in a single acquisition. In cadaveric studies, three-dimensional image registration achieved median target registration errors of 1.2 mm and 0.6° [26].

By capturing a larger anatomical volume in a single scan, 3DLS technology may improve surgical efficiency, reduce the need for repeat imaging, and support reliable intraoperative navigation and hardware verification. Additionally, the generation of larger, high-resolution imaging datasets may facilitate integration with emerging AI-based image processing and navigation platforms [25].

#### 3.3.3. Metal Artifact Reduction (O Arm)

Metal artifacts remain a persistent limitation of intraoperative CT imaging in spine surgery, frequently obscuring pedicle margins, vertebral boundaries, and adjacent neural structures—particularly in revision cases or when dense instrumentation is present. In 2019, Zhang et al. demonstrated that model-based “known-component” reconstruction techniques can substantially improve intraoperative image quality in the presence of spinal hardware [27]. Their pilot clinical study showed that this approach reduced metal-related blooming, decreasing screw-shaft overestimation by more than 60%, and minimized streak artifacts surrounding instrumentation. These improvements resulted in clearer visualization of pedicle boundaries and screw tips, as well as enhanced soft-tissue contrast compared with standard reconstruction methods [27].

Similarly, in 2020, Privalov et al. evaluated software-based metal artifact reduction (MAR) techniques for intraoperative cone-beam CT following pedicle screw placement [28]. They reported significant improvements in image quality, including reductions in screw-diameter overestimation and streaking artifacts [28].

Ongoing advancements in MAR technology have also been incorporated into commercially available platforms. For example, the Medtronic StealthStation 4.3 software update includes enhanced metal artifact reduction capabilities [25]. Although the specific reconstruction algorithms have not been publicly disclosed, these enhancements may improve detection of hardware malposition and support more precise intraoperative adjustments.

While dedicated studies linking MAR improvements to long-term clinical outcomes remain limited, enhanced image quality provides a critical foundation for the integration of more advanced computational tools, including AI-assisted imaging analysis. Improved intraoperative visualization of pedicle boundaries and screw tips may reduce the need for postoperative CT confirmation and facilitate immediate intraoperative revision, thereby improving operating room workflow efficiency; however, prospective data quantifying this impact on reoperation rates remain limited. Collectively, current evidence and emerging software developments suggest that advanced reconstruction technologies can meaningfully improve hardware assessment and intraoperative decision-making in spine surgery.

### 3.4. AI and AI-Adjacent Technology in Navigation and Registration

#### 3.4.1. Surface Landmarking to Preop CT (Stryker, Medtronic)

Surface-based registration using a preoperative CT dataset is a reliable option for image-guided spine surgery. This technique provides highly accurate alignment between the patient and the preoperative CT model without requiring intraoperative CT acquisition. Tamura et al. demonstrated that when surgeons register the patient to a preoperative CT scan using surface landmarking—by sampling approximately 20 points along the lamina—the system achieves submillimeter positional accuracy [29]. This level of precision makes the technique advantageous for navigation during pedicle screw placement. The authors emphasized that the method is practical because the lamina is routinely exposed during standard posterior approaches. Accuracy may be further enhanced by incorporating additional surface points along the sides of the spinous process, particularly in cases where laminar anatomy is distorted or limited, such as in revision surgery [29].

Importantly, single-time multilevel registration has been shown to permit accurate and safe implant placement. Postoperative CT validation of surface-based registration techniques has demonstrated excellent accuracy, with mean registration error reported at less than 1.0 mm, supporting precise pedicle screw placement and minimizing hardware-related complications and reoperations [30].

Despite these advantages, surface-based registration is not without limitations. Marzouk et al. reported significantly higher radiation exposure in patients undergoing preoperative CT followed by manual surface registration compared with those receiving intraoperative CT with automatic registration (median 424.50 mGy·cm for preoperative CT versus 317.00 mGy·cm for intraoperative CT), without significant differences in operative time [6]. Nevertheless, surface landmark registration remains an accessible and effective method for performing accurate image-guided spinal surgery.

#### 3.4.2. Optical Surface Landmarking to Preop CT (7D)

Machine-vision navigation represents a newer image-guided technology that utilizes structured light and optical topographic imaging to rapidly register a patient’s exposed bony anatomy to a preoperative CT dataset. Unlike traditional surface-based registration, which requires manual collection of discrete surface points, this system projects nonionizing patterned light onto the spine, captures a dense three-dimensional surface map, and automatically matches thousands of points to the preoperative CT scan within seconds.

This platform incorporates artificial intelligence in the form of machine-vision-based computer vision to recognize exposed bony structures and perform automated registration. Rather than relying on deep learning or predictive modeling, it employs rule-based image processing and pattern recognition algorithms to enable real-time navigation without the need for additional intraoperative ionizing radiation.

In the first reported clinical application of this system for unstable thoracolumbar fusion, Malacon et al. [31] demonstrated that the machine-vision navigation platform could register thousands of surface points in milliseconds and provide accurate CT-based navigation without repeated intraoperative imaging. The authors emphasized improvements in workflow efficiency, reduced susceptibility to inaccuracies caused by spinal movement relative to a reference clamp, and the ability to rapidly re-register when needed in unstable fracture cases [31].

Similarly, Stewart et al. performed a retrospective cohort study of 150 consecutive patients undergoing spinal instrumentation with the 7D visible light machine-vision navigation system [32]. There were no instances of symptomatic pedicle screw malposition, and no patients required reoperation or readmission related to screw placement. In a subset of cases, postoperative CT imaging confirmed appropriate screw positioning without pedicle breach. Use of the system was also associated with improved operative efficiency, including a 20% reduction in operative time for thoracolumbar deformity cases compared with C-arm-guided techniques, and an average registration plus bilateral screw placement time of 5 min and 25 s per level [32].

Taken together, these findings suggest that machine-vision navigation enhances workflow efficiency, reduces reliance on intraoperative radiation, mitigates inaccuracies related to reference frame movement, and allows rapid re-registration in complex or unstable cases.

#### 3.4.3. Two-Dimensional Fluoroscopy with Preop CT (Globus)

To bridge the gap between detailed preoperative anatomic planning and real-time intraoperative guidance, newer robot-assisted navigation workflows integrate preoperative CT imaging with intraoperative two-dimensional fluoroscopy to provide three-dimensional spatial context during instrumentation. In this approach, a high-resolution preoperative CT scan is used for surgical planning—including trajectory mapping and implant selection—while intraoperative fluoroscopic images are obtained after patient positioning to localize anatomy and establish registration between the patient and the preoperative dataset.

CT-to-fluoroscopy registration has been shown to be a feasible and accurate navigation strategy. In a multicenter retrospective analysis, Khan et al. reported high pedicle screw placement accuracy using CT-to-fluoroscopy registration with the Mazor X robotic system, with approximately 98% of screws graded as Gertzbein-Robbins A and the remainder graded as B, resulting in 100% clinically acceptable placement. Fluoroscopy exposure was comparable to alternative registration techniques [33].

Navigation platforms such as Globus Medical’s ExcelsiusGPS also support this workflow, enabling surgeons to leverage the detailed anatomic information provided by CT while maintaining familiar fluoroscopic guidance, without requiring routine intraoperative CT acquisition for every case. From a technical standpoint, CT-to-fluoroscopy navigation relies primarily on conventional image registration and tracking algorithms rather than artificial intelligence-based reconstruction or decision-support systems [34]. However, this approach may require longer initial setup time compared with traditional freehand or fluoroscopy-only techniques [33,34].

Overall, CT-to-fluoroscopy robotic navigation facilitates consistent pedicle screw trajectory planning and reduces reliance on repeated intraoperative fluoroscopic imaging. By leveraging preoperative CT data without requiring intraoperative CT acquisition, this approach may offer a more cost-accessible navigation alternative to dedicated O-arm-based workflows.

### 3.5. Emerging AI and AI-Adjacent Applications in Intraoperative Imaging

#### 3.5.1. Low Dose Intraoperative CT (O Arm/Smartdose)

Efforts to reduce radiation exposure during intraoperative three-dimensional imaging have demonstrated that substantial dose reductions can be achieved without compromising the evaluation of pedicle screw placement. In a cadaveric study by Sarwahi et al., reducing O-arm output to one-half and even one-third of the manufacturer’s recommended settings maintained screw assessment accuracy comparable to postoperative CT while significantly decreasing effective radiation dose [35].

This relationship between dose reduction and preserved imaging performance is consistent with the engineering framework underlying the FDA-cleared Spine Smart Dose (SSD) protocol [36]. Rather than acquiring nearly 400 projections, as in the standard O-arm 3D protocol, SSD collects approximately one-quarter of that number and compensates through a machine-learning-based reconstruction algorithm designed to mitigate image noise while maintaining diagnostic quality. According to FDA testing, SSD achieves an approximately 70% reduction in radiation dose compared with the standard acquisition protocol while producing images considered equivalent for clinical decision-making [36].

Collectively, the cadaveric data and SSD validation studies support the concept that modern intraoperative CT platforms can implement low-dose or “smart-dose,” protocols that meaningfully decrease patient radiation exposure while preserving navigation accuracy through AI-assisted reconstruction techniques.

Low-dose CT protocols are of particular importance in radiation-sensitive populations, including pediatric patients. To date, studies have demonstrated that low-dose intraoperative imaging provides image quality compatible with navigation software, supporting its integration into AI-assisted spine surgery workflows [37]. Furthermore, Carl et al. reported that when low-dose protocols were applied to intraoperative CT scanning for automatic registration, navigation accuracy was maintained across lumbar, thoracic, and cervical levels, indicating that dose reduction does not compromise registration performance [38].

Low-dose protocols may allow for easier implementation of CT-guided operative practice for physicians. Beyond radiation reduction, low-dose protocols may support broader adoption of intraoperative CT navigation in centers where cumulative patient dose is a regulatory or institutional constraint, without requiring hardware changes or additional acquisition time.

#### 3.5.2. Three-Dimensional Image Acquisition from 2D Imaging (X23D)

Recent advances in artificial intelligence have enabled reconstruction of three-dimensional spinal anatomy from limited intraoperative fluoroscopic imaging, addressing a key limitation of conventional two-dimensional X-ray guidance in spine surgery. Luchmann et al. described X23D, a deep learning-based method that estimates the three-dimensional shape of lumbar vertebrae using a sparse set of four intraoperatively acquired two-dimensional X-ray images, without requiring preoperative CT or cone-beam CT imaging [39]. This approach is particularly relevant in spine surgery, where accurate three-dimensional understanding of pedicle anatomy and vertebral orientation is critical for safe instrumentation, yet surgeons frequently rely on planar fluoroscopy with limited spatial feedback.

By incorporating X-ray calibration parameters directly into the reconstruction network, X23D preserves geometric fidelity while maintaining patient-specific anatomy, potentially reducing errors associated with mental three-dimensional interpretation of two-dimensional images. Reconstruction times of approximately four seconds suggest feasibility for intraoperative use, highlighting the potential of AI-based 2D-to-3D reconstruction to support navigation and intraoperative decision-making while decreasing reliance on traditional three-dimensional imaging modalities [39].

In an ex vivo laboratory study, the X23D navigation system was compared with conventional two-dimensional fluoroscopy-assisted freehand techniques and demonstrated similar accuracy with respect to pedicle breach rates and screw placement. Additionally, the system was associated with lower radiation doses and comparable reported user experience [39].

Overall, three-dimensional reconstruction from two-dimensional fluoroscopy, as exemplified by X23D technology, enables real-time 3D surgical navigation using standard intraoperative fluoroscopy. This capability offers the potential to reduce radiation exposure, minimize workflow disruption, and lower costs compared with conventional CT-based navigation systems.

#### 3.5.3. The Role of AI in MRI Based Navigation

Although CT-guided navigation remains the standard imaging modality for spinal instrumentation due to its superior visualization of osseous anatomy, it requires an additional imaging study that exposes patients to ionizing radiation. In contrast, MRI is routinely obtained as part of the preoperative workup and does not involve radiation exposure. The application of AI to generate synthetic CT (sCT) images from MRI therefore represents a potentially advantageous alternative for navigation. Several studies have evaluated this emerging technology. Morbée et al. demonstrated that quantitative morphometric measurements derived from MRI-based sCT of the lumbar spine, including key parameters of bony anatomy, were statistically equivalent to those obtained from conventional CT [40]. Similarly, Lafranca et al. performed a cadaveric study comparing AI-generated MRI-based sCT navigation to conventional CT for cervical pedicle screw placement [41]. Radiation-free sCT-guided navigation showed comparable safety and accuracy, with no significant differences in translational or angular deviation and similar rates of clinically acceptable screw placement according to Gertzbein-Robbins grading [41]. In a separate cadaveric study of thoracic and lumbar instrumentation using a scoliosis-specific MRI protocol, Lafranca et al. again demonstrated that AI-generated sCT allowed for pedicle screw planning and placement with accuracy comparable to CT-based navigation [42]. No clinically significant pedicle breaches (>2 mm) were observed, and translational and angular deviations between planned and final screw positions did not differ significantly between modalities [42]. Taken together, these findings support the feasibility of radiation-free, MRI-based spinal navigation using AI-generated sCT without compromising accuracy or safety in pedicle screw placement.

#### 3.5.4. The Role of AR and AI

Augmented reality (AR) is increasingly being explored as a complementary technology to artificial intelligence-driven imaging and navigation systems in spine surgery, enabling visualization of digital anatomical models and surgical guidance directly within the operative field.

De Jesus Encarnacion Ramirez et al. reported that AR provides three-dimensional visualization of spinal anatomy during surgery, improving intraoperative orientation and potentially reducing operative time while also offering benefits in surgical training and patient education [43]. Luigi-Martínez et al. described how AI techniques such as machine learning and computer vision can support automated image analysis and three-dimensional reconstruction, while AR platforms allow these data to be visualized intraoperatively to assist with implant positioning and surgical navigation [44]. Chatzis et al. highlighted the role of AI in improving surgical planning and outcome prediction in complex spine procedures, noting that integration with visualization technologies such as AR may further enhance intraoperative execution and support more personalized surgical strategies [45]. Together, these technologies represent an emerging approach to precision spine surgery, although further clinical validation is required.

#### 3.5.5. Implementation Challenges

Despite the technical promise of AI-integrated imaging and navigation platforms, several barriers must be addressed before widespread clinical adoption can be realized. Most AI-based imaging tools currently enter clinical use under FDA 510(k) clearance, a pathway that does not require prospective clinical trial evidence of superiority, resulting in many systems being deployed on the basis of cadaveric accuracy data or single-institution feasibility studies alone. Standardized performance benchmarks, including agreed-upon accuracy thresholds, breach grading criteria, and minimum validation cohort sizes, are needed for meaningful cross-platform comparison and regulatory oversight. Beyond validation, intraoperative CT systems represent substantial monetary investment, and reimbursement structures for AI-enhanced navigation remain inconsistent; hybrid workflows such as CT-to-fluoroscopy registration and AI-based 3D reconstruction may offer more accessible alternatives, though their long-term cost-effectiveness has not been formally evaluated. Training burden is an additional consideration, as efficiency gains with AI-augmented platforms appear to accrue after an initial adoption period, yet structured competency frameworks have not been standardized across systems or training programs. Finally, AI models trained on single-institution datasets may not generalize across scanner types, patient populations, or surgical workflows, and conditions common in spine surgery, including severe deformity, high BMI, and prior instrumentation, may degrade model performance if underrepresented in training data. The most pressing research gap remains the absence of prospective comparative trials evaluating whether AI-enhanced imaging technologies improve patient-centered outcomes, including complication rates, reoperation rates, operative time, and radiation exposure, relative to conventional navigation platforms.

### 3.6. Limitations and Future Directions

This review has several limitations. As a narrative review, literature selection was not performed using a formal systematic search strategy, introducing the potential for selection bias and incomplete capture of all relevant studies. The included evidence is heterogeneous, consisting of cadaveric investigations, small retrospective series, technical reports, and early feasibility studies, which limits direct comparison across platforms and constrains conclusions regarding clinical efficacy. A proportion of the available literature originates from industry-funded or manufacturer-sponsored validation studies, introducing potential for publication bias that independent replication has not yet fully addressed. Many AI-enabled platforms remain in early stages of validation with limited long-term outcome data, and rapid technological growth means available evidence may quickly become outdated as newer software iterations and imaging systems are introduced. Additionally, deep learning models developed at single institutions may not generalize across different scanner hardware, patient populations, or surgical workflows, and the opacity of black-box architectures complicates clinical interpretability and accountability. Data privacy, cybersecurity, and regulatory frameworks governing AI model updates in cleared devices represent further considerations as these systems mature. A full evaluation of cost-effectiveness, reimbursement, and institutional implementation strategies falls outside the scope of this review and represents an important direction for future health economics research. Despite these limitations, this review provides a comprehensive overview of the evolving role of AI in intraoperative spine surgery imaging and navigation, with the aim of informing clinical practice and guiding future investigation.

Future research should focus on strengthening the clinical evidence base for AI-enabled intraoperative imaging and navigation technologies. Many current studies remain limited to cadaveric validation, small retrospective cohorts, or single-center feasibility studies. Prospective multicenter investigations with standardized reporting of navigational accuracy, radiation exposure, and workflow metrics are needed to allow meaningful comparison across platforms. In addition, future work should evaluate patient-centered outcomes, including neurologic complications, reoperation rates, and functional recovery, to determine whether improvements in technical accuracy translate into measurable clinical benefit.

### 3.7. Summary

Current intraoperative imaging and navigation technologies exist at different stages of clinical maturity. Several modalities summarized in Table 1 are already widely used in clinical spine surgery, including intraoperative CT-based navigation systems such as O-arm and AIRO platforms, surface registration to preoperative CT, CT-to-fluoroscopy navigation used in robotic platforms, metal artifact reduction reconstruction techniques, and hardware improvements such as expanded CT scan windows. Optical surface landmarking systems have also demonstrated promising clinical accuracy and reduced radiation exposure, although their adoption remains institution-dependent. Emerging technologies entering clinical workflows include machine learning-assisted image analysis for pathology detection, low-dose intraoperative CT reconstruction protocols, and augmented reality-based surgical visualization, which have demonstrated promising early clinical feasibility but remain under ongoing evaluation. In contrast, several AI-driven approaches remain primarily investigational, including deep learning-based three-dimensional reconstruction of fluoroscopic images (e.g., X23D) and MRI-derived synthetic CT navigation. The relative clinical maturity and key characteristics of these technologies are summarized in Table 1.

## 4. Conclusions

Artificial intelligence is increasingly influencing multiple aspects of spine surgery, from diagnostic support to intraoperative imaging, navigation, and construct planning. Early applications in image and pathology recognition demonstrate that machine learning and deep learning can assist with identifying degenerative disease, stenosis, deformity, and traumatic injuries on spinal imaging, establishing an important foundation for AI-assisted surgical decision-making.

Intraoperatively, conventional imaging and navigation platforms—including intraoperative CT systems such as the O-arm, AIRO, and GE OEC 3D, surface-based registration to preoperative CT, and optical surface landmarking technologies such as 7D Surgical—remain the backbone of modern navigated spine surgery. Although these systems are not primarily AI-driven, they provide the high-quality imaging and registration accuracy required for safe instrumentation and decompression.

Several emerging technologies demonstrate how artificial intelligence may further optimize these workflows. Machine learning-based reconstruction algorithms have enabled low-dose intraoperative CT protocols that reduce radiation exposure while maintaining image quality. Expanded scan window capabilities improve visualization for long-segment constructs, while metal artifact reduction techniques enhance assessment of instrumentation in instrumented spines. Hybrid workflows combining 2D fluoroscopy with preoperative CT datasets offer navigation without routine intraoperative CT, and AI-based 3D reconstruction from limited 2D imaging introduces the possibility of real-time navigation with lower radiation and cost. Additionally, MRI-derived synthetic CT represents a promising radiation-free alternative for navigation while preserving accurate representation of osseous anatomy. 

## Figures and Tables

**Table 1:** Summary of AI applications in spine surgery imaging and navigation with reported performance metrics.

Modality/Technology	Imaging Source	AI Component	Study Type/Key References	Sample Size	Accuracy Metrics	Radiation Impact
Image recognition & pathology detection	Radiographs, CT, MRI	Machine learning/deep learning	Algorithm validation studies (Sanders et al. [16]; Veronezi et al. [17]; Pan et al. [18]; Hallinan et al. [19]; Doerr et al. [11])	Sanders et al. [16]: 250 Drawings Veronezi et al. [17]: 206 Radiographs Pan et al. [18] 500 Patients Hallinan et al. [19]: 446 MRIs Doerr et al. [11]: 111 patients	Sanders et al. [16]: 49% sensitivity Veronezi et al. [17]: 62.85% accuracy Pan et al. [18]: ~84–93% accuracy Hallinan et al. [19]: N/A; Doerr et al. [11]. up to 95.1% accuracy	Varies: Improved detection may lead to less repeat imaging
Intraoperative CT navigation (O-arm, AIRO, GE OEC)	Intraoperative CT	None (conventional navigation)	Clinical cohort (Miller et al. [20]; Rajasekaran et al. [21]; Costa et al. [23]; Zausinger et al. [24])	Miller et al. [20]: 240 screws Rajasekaran et al. [21]: 452 screws Costa et al. [23]: 11,144 screws Zausinger et al. [24]: 414 screws	Miller et al. [20]: 98.8% accuracy Rajasekaran et al. [21]: 96.2% Accuracy Costa et al. [23]: 98.5% accuracy Zausinger et al. [24]: 98.2% accuracy	May have higher intra-operative radiation exposure than fluoroscopy
Surface registration to preoperative CT	Preoperative CT + intraoperative landmarks	None (conventional navigation)	Clinical/technical studies (Tamura et al. [29]; Marzouk et al. [6])	Tamura et al. [29]: 20 surface points Marzouk et al. [6]: 480 screws	Tamura et al. [29]: Registration error < 1 mm	Requires preoperative CT Marzouk et al. [6]: 69% reduction in intraoperative X-ray radiation; 25% reduction in CT radiation
Optical surface landmarking (7D Surgical)	Optical surface mapping + preoperative CT	None (Computer-vision registration)	Case Report and cohort studies (Malacon et al. [31].; Stewart et al. [32])	Malacon et al. [31]: 1 patient Stewart et al. [32] 150 patients	No symptomatic screw malposition reported	Eliminates intraoperative radiation
Low-dose intraoperative CT protocols (SmartDose)	Intraoperative CT	ML-based reconstruction	Cadaveric and clinical validation (Sarwahi et al. [35]; Carl et al. [38])	Sarwahi et al. [35]: 8 Cadavers Carl et al. [38]: 645 Patients	N/A	Reduced radiation dose (~70% reduction reported)
Expanded scan window CT	Intraoperative CT	None (hardware improvement)	Phantom and cadaveric imaging studies (Zhang et al. [26])	Zhang et al. [26]: 25 screws	Target registration error ~1.2 mm and ~0.6°	Similar to standard CT acquisition
Metal artifact reduction (MAR)	CT imaging with instrumentation	None (Reconstruction Algorithms)	Imaging validation studies (Zhang et al. [27]; Privalov et al. [28])	Zhang et al. [27]: 17 Patients Privalov et al. [28]: 49 Patients	Zhang et al. [27]: 65.8% increase in attenuation accuracy Privalov et al. [28]: reduced the blooming artifacts significantly (*p* < 0.01)	No change in radiation dose
CT-to-fluoroscopy navigation (robotic systems)	Fluoroscopy + preoperative CT	None (conventional registration)	Multicenter retrospective clinical study (Khan et al. [33])	Khan et al. [33]: 268 Patients	Khan et al. [33]: ~98% screws graded Gertzbein-Robbins A	Eliminates intra-operative CT
3D reconstruction from 2D imaging (X23D)	Fluoroscopy	Deep learning	Experimental ex vivo study (Jecklin et al. [46])	Jecklin et al. [46]: 49 Screws	Jecklin et al. [46]: Comparable pedicle breach rates to fluoroscopy	Potential radiation reduction
MRI-derived synthetic CT navigation	MRI	Deep learning image synthesis	Imaging validation and cadaveric studies (Morbée et al. [40]; Lafranca et al. [41])	Morbée et al. [40]: 30 Patients Lafranca et al. [41]: 5 Cadavers	Morbée et al. [40]: No significant difference in screw deviation vs CT navigation Lafranca et al. [41]: 84% were grade A	Eliminates CT radiation
Augmented reality surgical visualization	CT or MRI datasets	AI-adjacent visualization	Narrative/technical studies (De Jesus Encarnacion Ramirez et al. [43]; Luigi-Martínez et al. [44]; Chatzis et al. [45])	De Jesus Encarnacion Ramirez et al. [43]: 11 studies Luigi-Martínez et al. [44]; Chatzis et al. [45]: Not applicable	Improved intraoperative visualization reported	No direct additional radiation

## Data Availability

No new data were created or analyzed in this study.

## References

[1] Yagi M., Yamanouchi K., Fujita N., Funao H., Ebata S. (2023). Revolutionizing Spinal Care: Current Applications and Future Directions of Artificial Intelligence and Machine Learning. J. Clin. Med..

[2] Crawford A.M., Striano B.M., Giberson-Chen C.C., Xiong G.X., Lightsey H.M., Schoenfeld A.J., Simpson A.K. (2023). Projected Lifetime Cancer Risk Associated with Intraoperative Computed Tomography for Lumbar Spine Surgery. Spine.

[3] Kendlbacher P., Tkatschenko D., Czabanka M., Bayerl S., Bohner G., Woitzik J., Vajkoczy P., Hecht N. (2022). Workflow and performance of intraoperative CT, cone-beam CT, and robotic cone-beam CT for spinal navigation in 503 consecutive patients. Neurosurg. Focus.

[4] Mason A., Paulsen R., Babuska J.M., Rajpal S., Burneikiene S., Nelson E.L., Villavicencio A.T. (2014). The accuracy of pedicle screw placement using intraoperative image guidance systems. J. Neurosurg. Spine.

[5] Zhao J., Liu Y., Fan M., Liu B., He D., Tian W. (2018). Comparison of the Clinical Accuracy Between Point-to-Point Registration and Auto-Registration Using an Active Infrared Navigation System. Spine.

[6] Marzouk M.M., Afghanyar Y., Marzouk M.M., Boussouf S.H., Hartung P., Richter M. (2022). Comparison of radiation exposure and surgery time between an intraoperative CT with automatic surface registration and a preoperative CT with manual surface registration in navigated spinal surgeries. Eur. Spine J..

[7] Feierabend M., Wolfgart J.M., Praster M., Danalache M., Migliorini F., Hofmann U.K. (2025). Applications of machine learning and deep learning in musculoskeletal medicine: A narrative review. Eur. J. Med. Res..

[8] Adida S., Legarreta A.D., Hudson J.S., McCarthy D., Andrews E., Shanahan R., Taori S., Lavadi R.S., Buell T., Hamilton D.K. (2024). Machine Learning in Spine Surgery: A Narrative Review. Neurosurgery.

[9] Wu H., Bailey C., Rasoulinejad P., Li S. (2018). Automated comprehensive Adolescent Idiopathic Scoliosis assessment using MVC-Net. Med. Image Anal..

[10] Rosenberg G.S., Cina A., Schiró G.R., Giorgi P.D., Gueorguiev B., Alini M., Varga P., Galbusera F., Gallazzi E. (2022). Artificial Intelligence Accurately Detects Traumatic Thoracolumbar Fractures on Sagittal Radiographs. Medicina.

[11] Doerr S.A., Weber-Levine C., Hersh A.M., Awosika T., Judy B., Jin Y., Raj D., Liu A., Lubelski D., Jones C.K. (2022). Automated prediction of the Thoracolumbar Injury Classification and Severity Score from CT using a novel deep learning algorithm. Neurosurg. Focus.

[12] Nam K.H., Seo I., Kim D.H., Lee JIl Choi B.K., Han I.H. (2019). Machine Learning Model to Predict Osteoporotic Spine with Hounsfield Units on Lumbar Computed Tomography. J. Korean Neurosurg. Soc..

[13] Grob A., Loibl M., Jamaludin A., Winklhofer S., Fairbank J.C., Fekete T., Porchet F., Mannion A.F. (2022). External validation of the deep learning system “SpineNet” for grading radiological features of degeneration on MRIs of the lumbar spine. Eur. Spine J..

[14] Guha D., Jakubovic R., Alotaibi N.M., Klostranec J.M., Saini S., Deorajh R., Gupta S., Fehlings M.G., Mainprize T.G., Yee A. (2019). Optical Topographic Imaging for Spinal Intraoperative Three-Dimensional Navigation in Mini-Open Approaches: A Prospective Cohort Study of Initial Preclinical and Clinical Feasibility. World Neurosurg..

[15] Burström G., Buerger C., Hoppenbrouwers J., Nachabe R., Lorenz C., Babic D., Homan R., Racadio J.M., Grass M., Persson O. (2019). Machine learning for automated 3-dimensional segmentation of the spine and suggested placement of pedicle screws based on intraoperative cone-beam computer tomography. J. Neurosurg. Spine.

[16] Sanders N.W., Mann N.H. (2000). Automated scoring of patient pain drawings using artificial neural networks: Efforts toward a low back pain triage application. Comput. Biol. Med..

[17] Veronezi C.C.D., Simões P.W.T.D.A., Santos R.L.D., Rocha E.L.D., Melão S., Mattos M.C.D., Cechinel C. (2011). Computational analysis based on artificial neural networks for aiding in diagnosing osteoarthritis of the lumbar spine. Rev. Bras. Ortop. (Engl. Ed.).

[18] Pan Q., Zhang K., He L., Dong Z., Zhang L., Wu X., Wu Y., Gao Y. (2021). Automatically Diagnosing Disk Bulge and Disk Herniation with Lumbar Magnetic Resonance Images by Using Deep Convolutional Neural Networks: Method Development Study. JMIR Med. Inform..

[19] Hallinan J.T.P.D., Zhu L., Yang K., Makmur A., Algazwi D.A.R., Thian Y.L., Lau S., Choo S., Eide S.E., Yap Q.V. (2021). Deep Learning Model for Automated Detection and Classification of Central Canal, Lateral Recess, and Neural Foraminal Stenosis at Lumbar Spine MRI. Radiology.

[20] Miller C.A., Ledonio C.G., Hunt M.A., Siddiq F., Polly D.W. (2016). Reliability of the Planned Pedicle Screw Trajectory versus the Actual Pedicle Screw Trajectory using Intra-operative 3D CT and Image Guidance. Int. J. Spine Surg..

[21] Rajasekaran S., Bhushan M., Aiyer S., Kanna R., Shetty A.P. (2018). Accuracy of pedicle screw insertion by AIRO^®^ intraoperative CT in complex spinal deformity assessed by a new classification based on technical complexity of screw insertion. Eur. Spine J..

[22] GE HealthCare (2024). Routine CBCT Imaging in Spine Surgery with OEC 3D. https://www.gehealthcare.co.uk/insights/article/routine-cbct-imaging-in-spine-surgery-with-oec-3d.

[23] Costa F., Dorelli G., Ortolina A., Cardia A., Attuati L., Tomei M., Milani D., Balzarini L., Galbusera F., Morenghi E. (2015). Computed Tomography-Based Image-Guided System in Spinal Surgery. Oper. Neurosurg..

[24] Zausinger S., Scheder B., Uhl E., Heigl T., Morhard D., Tonn J.C. (2009). Intraoperative Computed Tomography with Integrated Navigation System in Spinal Stabilizations. Spine.

[25] Medtronic O-arm^TM^ Surgical Imaging System. https://europe.medtronic.com/content/dam/medtronic-wide/public/western-europe/medical-specialties/spine-surgery/o-arm-4-3-o2-brochure-en.pdf.

[26] Zhang X., Uneri A., Wu P., Ketcha M.D., Jones C.K., Huang Y., Larry Lo S.F., Helm P.A., Siewerdsen J.H. (2021). Long-length tomosynthesis and 3D-2D registration for intraoperative assessment of spine instrumentation. Phys. Med. Biol..

[27] Zhang X., Uneri A., Webster Stayman J., Zygourakis C.C., Lo S.F.L., Theodore N., Siewerdsen J.H. (2019). Known-component 3D image reconstruction for improved intraoperative imaging in spine surgery: A clinical pilot study. Med. Phys..

[28] Privalov M., Mohr M., Swartman B., Beisemann N., Keil H., Franke J., Grützner P.A., Vetter S.Y. (2020). Evaluation of Software-Based Metal Artifact Reduction in Intraoperative 3D Imaging of the Spine Using a Mobile Cone Beam CT. J. Digit. Imaging.

[29] Tamura Y., Sugano N., Sasama T., Sato Y., Tamura S., Yonenobu K., Yoshikawa H., Ochi T. (2005). Surface-based registration accuracy of CT-based image-guided spine surgery. Eur. Spine J..

[30] Papadopoulos E.C., Girardi F.P., Sama A., Sandhu H.S., Cammisa F.P. (2005). Accuracy of single-time, multilevel registration in image-guided spinal surgery. Spine J..

[31] Malacon K., Fatemi P., Zygourakis C.C. (2022). First reported use of machine vision image guided system for unstable thoracolumbar fusion: Technical case report. Interdiscip. Neurosurg..

[32] Stewart G. (2022). Visible Light Navigation in Spine Surgery: My Experience with My First 150 Cases. Int. J. Spine Surg..

[33] Khan A., Soliman M.A., Lee N.J., Waqas M., Lombardi J.M., Boddapati V., Levy L.C., Mao J.Z., Park P.J., Mathew J. (2022). CT-to-fluoroscopy registration versus scan-and-plan registration for robot-assisted insertion of lumbar pedicle screws. Neurosurg. Focus.

[34] Vaccaro A.R., Harris J.A., Hussain M.M., Wadhwa R., Chang V.W., Schroerlucke S.R., Samora W.P., Passias P.G., Patel R.D., Panchai R.R. (2020). Assessment of Surgical Procedural Time, Pedicle Screw Accuracy, and Clinician Radiation Exposure of a Novel Robotic Navigation System Compared with Conventional Open and Percutaneous Freehand Techniques: A Cadaveric Investigation. Global Spine J..

[35] Sarwahi V., Payares M., Wendolowski S., Maguire K., Thornhill B., Lo Y., Amaral T.D. (2017). Low-Dose Radiation 3D Intraoperative Imaging: How Low Can We Go? An O-Arm, CT Scan, Cadaveric Study. Spine.

[36] U.S. Food and Drug Administration (2024). O-Arm O2 Imaging System, version 4.3.0; 510(k) Premarket Notification (K240465).

[37] Sensakovic W.F., O’Dell M.C., Agha A., Woo R., Varich L. (2017). CT Radiation Dose Reduction in Robot-assisted Pediatric Spinal Surgery. Spine.

[38] Carl B., Bopp M., Saß B., Pojskic M., Gjorgjevski M., Voellger B., Nimsky C. (2019). Reliable navigation registration in cranial and spine surgery based on intraoperative computed tomography. Neurosurg. Focus.

[39] Luchmann D., Jecklin S., Cavalcanti N.A., Laux C.J., Massalimova A., Esfandiari H., Farshad M., Fürnstahl P. (2024). Spinal navigation with AI-driven 3D-reconstruction of fluoroscopy images: An ex-vivo feasibility study. BMC Musculoskelet. Disord..

[40] Morbée L., Chen M., Herregods N., Pullens P., Jans L.B.O. (2021). MRI-based synthetic CT of the lumbar spine: Geometric measurements for surgery planning in comparison with CT. Eur. J. Radiol..

[41] Lafranca P.P., Rommelspacher Y., Muijs S., Walter S.G., van der Velden T.A., Castelein R.M., Ito K., Seevinck P.R., Schlösser T.P. (2025). Safety and accuracy of cervical pedicle screw navigation using artificial intelligence–generated, MRI-based synthetic CT versus conventional CT. J. Neurosurg. Spine.

[42] Lafranca P.P., Rommelspacher Y., Walter S.G., Muijs S.P., van der Velden T.A., Shcherbakova Y.M., Castelein R.M., Ito K., Seevinck P.R., Schlösser T.P. (2026). The safety and accuracy of radiation-free spinal navigation using a short, scoliosis-specific BoneMRI-protocol, compared to CT. Eur. Spine J..

[43] De Jesus Encarnacion Ramirez M., Chmutin G., Nurmukhametov R., Soto G.R., Kannan S., Piavchenko G., Nikolenko V., Efe I.E., Romero A.R., Mukengeshay J.N. (2024). Integrating Augmented Reality in Spine Surgery: Redefining Precision with New Technologies. Brain Sci..

[44] Luigi-Martínez H.E., Layuno-Matos J.G., Fernández-Vélez N.A., Fernández-Soltero R., Señeriz-Ortiz R. (2025). Artificial Intelligence and Augmented Reality in Orthopedic Surgery: A Narrative Review of Current Applications and Future Directions. Cureus.

[45] Chatzis K.D., Tretiakov P., Passias P.G. (2025). Implementation of artificial intelligence (AI) in ASD treatment. N. Am. Spine Soc. J. (NASSJ).

[46] Jecklin S., Jancik C., Farshad M., Fürnstahl P., Esfandiari H. (2022). X23D—Intraoperative 3D Lumbar Spine Shape Reconstruction Based on Sparse Multi-View X-ray Data. J. Imaging.

